# Supplementary material for: Acceptance and attitudes toward COVID-19 vaccines: A cross-sectional study from Jordan
Source: PLoS One. 2021 Apr 23;16(4):e0250555. doi: 10.1371/journal.pone.0250555 (PMC8064595; doi:10.1371/journal.pone.0250555)
Supplement: S1 Table — (DOCX) [file pone.0250555.s001.docx]

**Acceptance and attitudes toward COVID-19 vaccines: A cross-sectional study from Jordan**

Tamam El-Elimat, Mahmoud M. AbuAlSamen, Basima A. Almomani, Nour A. Al-Sawalha, and Feras Q. Alali

| **S1 Table. The Final Version of the Questionnaire in Arabic and English.** | | |
| --- | --- | --- |
| **Demographics** | | |
| **المعلومات الديموغرافية** | | |
| **Gender** | 🞎 Male 🞎 Female | |
| 🞎 ذكر 🞎 أنثى | | **الجنس** |
| **Age (years)** | **_ _ _ _ _ _ _ _** | |
| **_ _ _ _ _ _ _ _** | | **العمر (بالسنوات)** |
| **Marital status** | 🞎 Single 🞎 Married 🞎 Others | |
|  | |  |
| 🞎 أعزب 🞎 متزوج 🞎 غير ذلك | | **الحالة الإجتماعية** |
| **Do you have kids?** | 🞎 Yes 🞎 No | |
| 🞎 نعم 🞎 لا | | **هل لديك ابناء؟** |
| **Education (Degree)** | 🞎 School education 🞎 Diploma  🞎 Undergraduate 🞎 Postgraduate | |
| 🞎 المرحلة الاساسية او الثانوية 🞎 دبلوم  🞎 بكالوريوس 🞎 دراسات عليا | | **أعلى درجة علمية؟** |
| **Educational background** | 🞎 Health-related 🞎 Non-health related | |
| 🞎 تخصص طبي 🞎 تخصص غير طبي | | **المجال التعليمي** |
| **Employment** | 🞎 Employed 🞎 Unemployed 🞎 Retired | |
| 🞎 على رأس عملي 🞎 لا أعمل 🞎 متقاعد | | **الوضع الوظيفي** |
| **Nationality** | 🞎 Jordanian 🞎 Non-Jordanian | |
| 🞎 أردنية 🞎 غير أردنية | | **الجنسية** |
| **Monthly income** | 🞎 <250 JD 🞎 250-599 JD 🞎 600-1000 🞎 >1000 JD | |
| 🞎 أقل من 250 🞎 250-599  🞎 600-1000 🞎 أكثر من 1000 دينار | | **الدخل الشهري للفرد (بالدينار الأردني)** |
| **Health insurance coverage** | 🞎 Yes, I have a governmental /military health insurance  🞎 Yes, I have a private health insurance  🞎 No, I do not have a health insurance | |
| 🞎 تأمين صحي حكومي أو خدمات طبية  🞎 تأمين صحي خاص  🞎 بدون تأمين صحي | | **التأمين الصحي** |
| **Smoking status** | 🞎 Current smoker (cigarettes, e-cigarettes, Shisha (Argila)  🞎 Ex-smoker (cigarettes, e-cigarettes, Shisha (Argila)  🞎 Not a smoker | |
| 🞎 مدخن (سجائر، سجائر الكترونية، ارجيلة)  🞎 مدخن سابق (سجائر، سجائر الكترونية، ارجيلة)  🞎 غير مدخن | | **التدخين** |
| **Medical History** | | |
| **التاريخ المرضي** | | |
| **Do you suffer from chronic diseases?** | 🞎 Yes 🞎 No | |
| 🞎 نعم 🞎 لا | | **هل تعاني من أي أمراض مزمنة؟** |
| **Attitudes towards Vaccines and Immunization** | | |
| **اتجاهات المرضى نحو المطاعيم والمناعة** | | |
| **In general, vaccines are safe.** | 🞎 Strongly agree 🞎 Agree 🞎 Neutral  🞎 Disagree 🞎 Strongly disagree | |
| 🞎 أتفق بشدة 🞎 أتفق 🞎 محايد 🞎 لا أتفق 🞎 لا أتفق بشدة | | **تعتبر المطاعيم بشكل عام آمنة.** |
| **Have you or someone you know ever had a bad reaction to a vaccine?** | 🞎 Yes 🞎 No 🞎 Not sure | |
| 🞎 نعم 🞎 لا 🞎 غير متأكد | | **هل تعرضت أنت أو أي شخص تعرفه لآثار جانبية سيئة ناتجة عن تناول مطاعيم؟** |
| **Have you received the influenza vaccine?** | 🞎 Yes, I took it this year  🞎 Yes, I took it last year  🞎 No, I did not take it this year  🞎 No, I did not take it last year | |
| 🞎 نعم تناولته هذه السنة  🞎 نعم تناولته السنة الماضية  🞎لا لم اتناوله هذه السنة  🞎 لا لم اتناوله السنة الماضية | | **هل تناولت مطعوم الانفلونزا الموسمية السنة الماضية؟** |
| **COVID-19 Pandemic** | | |
| **جائحة فيروس كورونا (كوفيد-19)** | | |
| **Who of the following tested positive for COVID-19?** | 🞎 Myself  🞎 A family member  🞎 A friend  🞎 Colleague  🞎 A neighbor  🞎 No one | |
| 🞎 أنا شخصياً  🞎 أحد افراد عائلتي  🞎 أحد أصدقائي  🞎 أحد زملائي في العمل  🞎 أحد جيراني  🞎 لا أحد | | **أي ممن يلي ثبُت إصابتهم بفيروس كورونا من خلال الفحص المخبري؟؟** |
| **Do you think that you may have been exposed to or infected with COVID-19 (without testing)?** | 🞎 Yes 🞎 No | |
| 🞎 نعم 🞎 لا | | **هل تعتقد أنك ربما تعرضت أو أصبت بفيروس كورونا (كوفيد-19) (بدون اختبار)؟** |
| **What are you most worried about during this COVID-19 pandemic? (Tick not more than 5)** | 🞎 Fear of becoming infected myself  🞎 Fear of a family member becoming infected  🞎 Death  🞎 Financial related worries  🞎 Job-related worries  🞎 Food insecurity related worries  🞎 Unavailability of vaccines  🞎 Being a plot or conspiracy  🞎 Being forced to take a medication  🞎 Being forced to take a vaccine  🞎 I am not worried about any issues  🞎 Other | |
| 🞎 الخوف من أن أصاب بالفيروس  🞎 الخوف من إصابة أحد أفراد الأسرة بالعدوى  🞎 الموت  🞎 مخاوف مالية  🞎 مخاوف متعلقة بالعمل  🞎 عدم توافر مطعوم للفيروس  🞎 أن تكون هناك مؤامرة  🞎 إجباري على تناول دواء للفيروس  🞎 إجباري على تناول مطعوم  🞎 أنا لست قلقاً بشأن أي مشكلة  🞎 أخرى | | **ما أكثر ما يقلقك خلال جائحة كورونا (كوفيد-19)؟ (ضع علامة على كل ما ينطبق)** |
| **Perspectives Toward COVID-19 Vaccination** | | |
| **وجهات نظر بخصوص مطعوم فيروس كورونا (كوفيد-19)** | | |
| **Do you think it is important to get a vaccine to protect the people from COVID-19?** | 🞎 Strongly agree 🞎 Agree 🞎 Neutral  🞎 Disagree 🞎 Strongly disagree | |
| 🞎 أتفق بشدة 🞎 أتفق 🞎 محايد 🞎 لا أتفق 🞎 لا أتفق بشدة | | **هل تعتقد أنه من المهم الحصول على مطعوم لحماية الناس من فيروس كورونا (كوفيد-19)؟** |
| **Pharmaceutical companies are going to develop safe and effective COVID-19 vaccines?** | 🞎 Strongly agree 🞎 Agree 🞎 Neutral  🞎 Disagree 🞎 Strongly disagree | |
| 🞎 أتفق بشدة 🞎 أتفق 🞎 محايد 🞎 لا أتفق 🞎 لا أتفق بشدة | | **ستقوم شركات الأدوية بتطوير مطاعيم آمنة وفعالة لمواجهة فيروس كورونا.** |
| **Do you believe COVID-19 vaccines made in Europe or America are safer than those made in other world countries?** | 🞎 Strongly agree 🞎 Agree 🞎 Neutral  🞎 Disagree 🞎 Strongly disagree | |
| 🞎 أتفق بشدة 🞎 أتفق 🞎 محايد 🞎 لا أتفق 🞎 لا أتفق بشدة | | **هل تعتقد أن مطاعيم فيروس كورونا (كوفيد-19) المصنعة في أوروبا أو أمريكا أكثر أماناً من تلك المصنعة في دول العالم الأخرى؟** |
| **I would take a vaccine to protect against COVID-19 once becomes available.** | 🞎 Strongly agree 🞎 Agree 🞎 Neutral  🞎 Disagree 🞎 Strongly disagree | |
| 🞎 أتفق بشدة 🞎 أتفق 🞎 محايد 🞎 لا أتفق 🞎 لا أتفق بشدة | | **سأتناول مطعوم للوقاية من فيروس كورونا عند توفره.** |
| **My concerns about related side effects will prevent me from taking a vaccine for the prevention of COVID-19.** | | 🞎 Strongly agree 🞎 Agree 🞎 Neutral  🞎 Disagree 🞎 Strongly disagree |
| 🞎 أتفق بشدة 🞎 أتفق 🞎 محايد 🞎 لا أتفق 🞎 لا أتفق بشدة | | **ستمنعني مخاوفي بشأن الآثار الجانبية من تناول مطعوم للوقاية من فيروس كورونا.** |
| **Most people will refuse to take the COVID-19 vaccine once licensed in Jordan.** | 🞎 Strongly agree 🞎 Agree 🞎 Neutral  🞎 Disagree 🞎 Strongly disagree | |
| 🞎 أتفق بشدة 🞎 أتفق 🞎 محايد 🞎 لا أتفق 🞎 لا أتفق بشدة | | **سيرفض معظم المواطنين تناول مطعوم لفيروس كورونا (كوفيد-19) بمجرد الموافقة على اجازته في الأردن.** |
| **The government will make the vaccine available for all citizens for free?** | 🞎 Strongly agree 🞎 Agree 🞎 Neutral  🞎 Disagree 🞎 Strongly disagree | |
| 🞎 أتفق بشدة 🞎 أتفق 🞎 محايد 🞎 لا أتفق 🞎 لا أتفق بشدة | | **ستوفر الحكومة مطعوم فيروس كورونا لجميع المواطنين مجاناً.** |
| **Would you be willing to pay for a COVID-19 vaccine privately?** | 🞎 Yes 🞎 No 🞎 Not sure | |
| 🞎 نعم 🞎 لا 🞎 غير متأكد | | **هل انت مستعد لدفع ثمن مطعوم فيروس كورونا (كوفيد-19) من مالك الخاص؟** |
| **Who do you trust the most for information about vaccines?** | 🞎 Media (TV, Radio, Newspaper)  🞎 Internet  🞎 Social media (Facebook, Twitter, WhatsApp, ..etc.)  🞎 Health care providers: Physicians, pharmacists, etc.  🞎 Family-members  🞎 Government (JFDA)  🞎 The pharmaceutical company reports  🞎 Scientific articles  🞎 I do not trust any source | |
| 🞎 وسائل الإعلام (تلفزيون ، راديو ، جريدة)  🞎 الإنترنت  🞎 وسائل التواصل الاجتماعي (فيس بوك، تويتر، واتساب .. الخ)  🞎 مقدمو الرعاية الصحية: الأطباء والصيادلة ، إلخ.  🞎 أفراد الأسرة  🞎 الحكومة ممثلة بالمؤسسة العامة للغذاء والدواء  🞎 تقارير شركات الأدوية  🞎 الدوريات العلمية  🞎 أنا لا أثق في أي مصدر | | **ما هي مصادر المعلومات الأكثر موثوقية بالنسبة لك للحصول على معلومات حول المطاعيم؟** |
